# Physician report cards and rankings yield long-lasting hand hygiene compliance exceeding 90 %

**DOI:** 10.1186/s13054-015-1008-4

**Published:** 2015-08-14

**Authors:** John Adam Reich, Monica E. Goodstein, Susan E. Callahan, Kathleen M. Callahan, Lindsay W. Crossley, Shira I. Doron, David R. Snydman, Stanley A. Nasraway

**Affiliations:** Department of Anesthesiology, Tufts Medical Center and the Tufts University School of Medicine, 800 Washington Street, Boston, MA 02111 USA; Department of Surgery, Tufts Medical Center and the Tufts University School of Medicine, 800 Washington Street, Boston, MA 02111 USA; Department of Nursing, Tufts Medical Center and the Tufts University School of Medicine, 800 Washington Street, Boston, MA 02111 USA; Division of Infectious Diseases, Tufts Medical Center and the Tufts University School of Medicine, 800 Washington Street, Boston, MA 02111 USA

## Abstract

**Introduction:**

Hand hygiene is an effective, low-cost intervention that prevents the spread of multidrug-resistant bacteria. Despite mandatory education and reminders, compliance by physicians in our hospital remained stubbornly low. Our objective was to study whether surveillance by our unit coordinator (secretary) paired with regular feedback to chiefs of service would increase physician hand hygiene compliance in the ICU.

**Method:**

The ICU unit coordinator was trained to observe and measure hand hygiene compliance. Data were collected on hand hygiene compliance at room entry and exit for 9 months. Percentage compliance for each medical and surgical subspecialty was reported to chiefs of service at the end of each month. Comparative rankings by service were widely distributed throughout the physician organization and the medical center.

**Results:**

The hand hygiene compliance rate among physicians increased from 65.1 % to 91.6 % during the study period (*p* <0.0001). More importantly in the succeeding 24 months after study completion, physician hand hygiene compliance remained >90 % in every month.

**Conclusions:**

Physician hand hygiene compliance increased as a consequence of the surveillance conducted by a full-time ICU team member, leading to a highly significant increase in the number of observations. In turn, this allowed for specific comparative monthly feedback to individual chiefs of service. Over the next 2 years after the study ended, these gains were sustained, suggesting an enduring culture change in physician behavior.

## Introduction

Intensive care unit (ICU)-acquired infection is directly related to hospital mortality [1]. Healthcare-associated infections (HCAI) are estimated at nearly 2 million infections per year and cause 98,000 preventable deaths per year in the US alone [1]. Hand hygiene is an effective, low-cost intervention that can prevent the spread of bacterial pathogens, including multidrug-resistant organisms such as methicillin-resistant *Staphylococcus aureus* (MRSA) [2]. Improvements in hand hygiene have been shown to decrease rates of HCAI [3–5].

Guidelines stress the importance of performing hand hygiene before and after all patient and equipment contact [6, 7]. The Joint Commission has also made hand hygiene compliance a high-priority initiative [8]. A systematic review found that compliance was lower in ICUs (30–40 %) than in other settings (40–50 %), and that physicians (32 %) were less compliant than nurses (48 %) [9]. Certain task-oriented specialties, such as anesthesiology, surgery and critical care medicine, have especially low rates of compliance [10, 11]. Prior to this study a comprehensive program and strong commitment by our hospital leadership resulted in rates of hand hygiene compliance, which exceeded the institutional goal of 90 % [12]. This program includes mandatory education and placement of alcohol-based hand rub (ABHR) in accessible positions outside the door of every room. Unfortunately, this level of compliance was not sustained, necessitating a reinvigorated approach to improve hand hygiene compliance.

The purpose of this study was to increase and sustain physician hand hygiene compliance to ≥90 % in the surgical intensive care unit (SICU). Hospital policy, approved by the Infection Prevention Committee, mandates hand hygiene to be performed upon room entry and room exit (“pump in, pump out”) using a 63 % isopropyl alcohol-based hand wash or water and antibacterial soap. Room entry and exit, i.e., “pump in, pump out” is not a specific hand hygiene indication [7]. However, in a medical center survey entry and exit have been identified as significant opportunities for hand hygiene [13]. We chose to measure this because it is our hospital policy, because it did not violate the patient’s privacy, and could be observed from the central unit coordinator’s desk. There is no one best way to measure hand hygiene; all have their advantages and disadvantages [14]. In January 2012, computerized physician order entry (CPOE) was adopted in the SICU. The SICU unit coordinator was thus substantially liberated from manually scanning, faxing, or entering orders. By assigning our unit coordinator to hand hygiene observations, large amounts of hand hygiene compliance data suddenly became obtainable. We hypothesized that giving individual physician chiefs detailed and extensive feedback monthly would significantly increase hand hygiene compliance.

## Methods

### Setting, study population and design

Tufts Medical Center is a 415-bed, level 1 trauma tertiary-care medical center in Boston, Massachusetts. The Tufts Medical Center SICU is a 10-bed unit that accepts a diverse cohort of noncardiac surgical patients from more than ten different surgical specialties. Attending intensivists round daily and lead a team of house staff and medical students. The unit is semiclosed meaning that surgical teams see their patients daily, while the SICU team is the primary managing team in the unit.

Infection prevention nurses trained the SICU coordinator according to written guidelines for hand hygiene observations [8]. In this role, she recorded hand hygiene compliance as part of her regular duties during her 40-hour workweek from 6 am to 2 pm Monday through Friday. Healthcare workers (HCWs) were identified by classification, level of training, and physician team. Approval from Tufts Health Science Institutional Review Board (IRB) was sought, and its decision was that no informed consent was needed as this quality improvement project was classified as nonhuman subject research. There were no ethical conflicts of interest concerning to the IRB.

Data were collected prospectively from March (month 1) to November (month 9) of 2012 on a daily basis by the unit coordinator, who directly observed providers exiting and entering patient rooms in the 10-bed SICU. There were no other new infection prevention educational programs or initiatives introduced during this period. Compliance was defined as the number of times hand hygiene was correctly performed upon either entering or exiting the room. A compliant encounter was one where the provider pumped ABHR while crossing the threshold of the room. A noncompliant encounter was recorded if a provider crossed the threshold of the room without pumping with ABHR. Each time a person crossed the threshold of the room counted as an encounter and was scored compliant or noncompliant; entrance and exit of the room were not paired as a single encounter but scored separately. Providers were not informed of the active surveillance but it became widely known over time that the unit coordinator was recording data.

Hand hygiene compliance rates were distributed via a monthly email report card to chiefs of services and presented monthly at Critical Care Committee and Infection Prevention meetings. The email report card contained a ranking list for each service from most compliant to least compliant. When physician chiefs questioned the authenticity of the rankings related to their service, they were provided the raw data by spreadsheet.

Hand hygiene compliance data for physicians were compiled into 9-monthly compliance percentages, plotted by scatter plot and fit with linear (least squares) regression and *P* value was determined for the slope. In addition, we collected compliance rates for 24 months (2013–2014) following completion of this study to assess sustainability in spite of not providing feedback. Authors report no conflicts of interests.

## Results

The unit coordinator made a total of 14,671 observations of physicians, house staff, and physician assistants over the 9-month study period. This number of observations in our ten-bed SICU was more than the aggregate number of hand hygiene encounters of physicians submitted by the entire rest of the hospital during this study period. For perspective, only 206 observations of this same physician group were reported from the SICU in the entire calendar year of 2011, of which the aggregate compliance rate was 77 %.

**Fig. 1:**
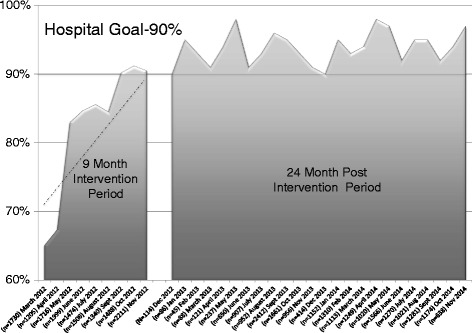
Hand hygiene compliance trend in intervention group and post intervention group. Physicians’ rate of change (slope) was 3.1 % per month (*p* <0.001 95 % confidence interval (CI) 1.65–4.60) during the time when feedback was provided with report cards. Sustained compliance rate greater than 90 % is shown in the 24-month post study period

**Fig. 2:**
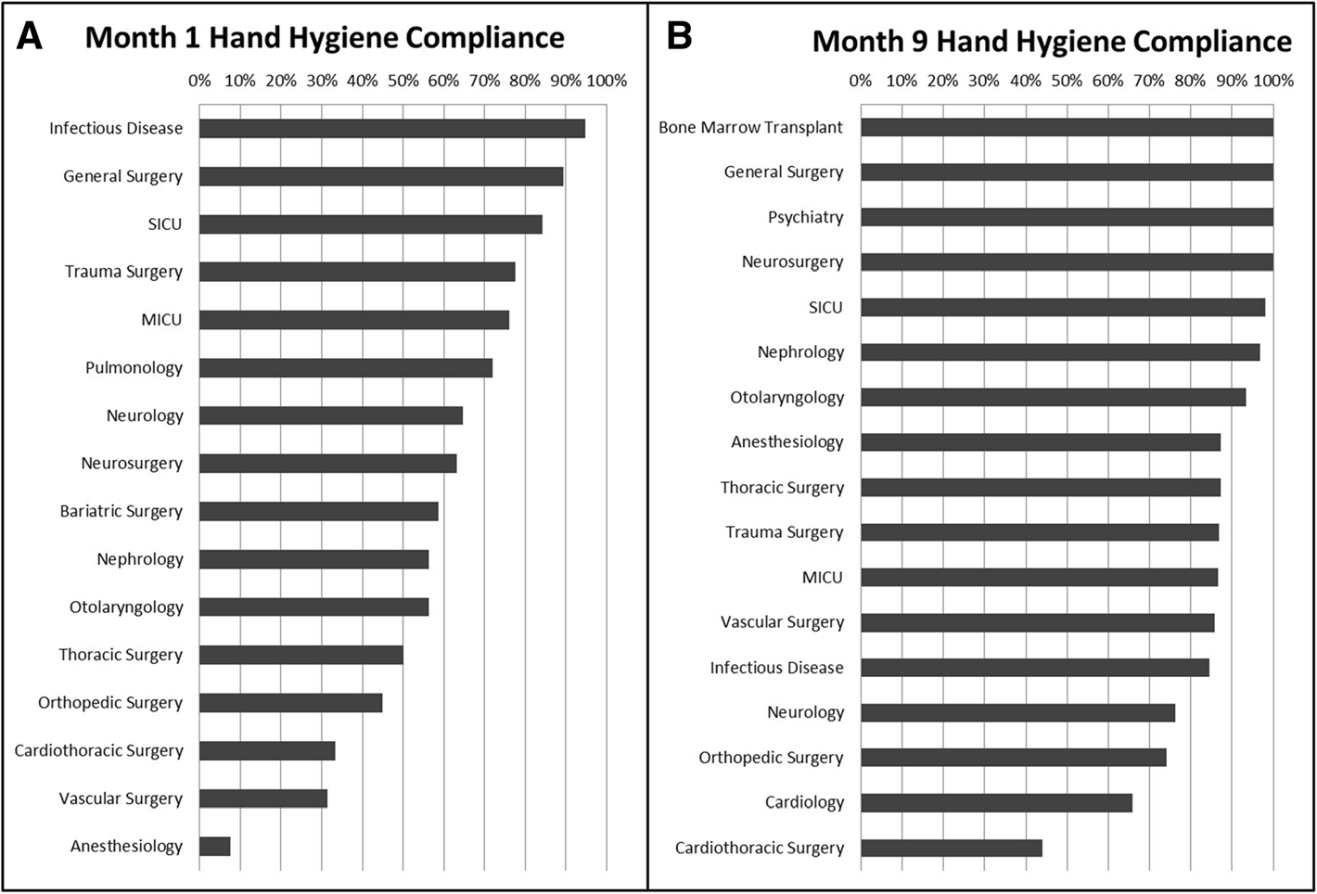
Panel A is hand hygiene compliance at beginning of study by specialty. Panel B is hand hygiene compliance at end of the study by specialty. Specialties are ranked from highest to lowest compliance. These graphs were emailed monthly to chiefs of service and demonstrate their ranking in relation to other services

An overall hand hygiene compliance rate of 83.3 % was observed during the 9-month study period. Physicians increased their compliance rate from 65.1 % in the first month to 91.2 % in the last month. The slope of the regression curve represents the average rate of change for physicians of 3.1 % per month (*p* <0.0001) with 95 % confidence interval 1.65–4.60 %. The SICU team, trauma surgery service, and neurosurgery services combined to have more than 60 % of the total physician observations. Physicians met the institutional target goal rate of greater than 90 % in each of the final 3 months of the study and in each month of the ensuing 24-month post study period when no feedback was given to chiefs of service (Fig. 1). Examples of monthly feedback provided to chiefs of service are shown for study months 1 and 9 in Fig. 2.

To be sure those differences were true and not specifically related to outliers, a sensitivity analysis was performed and regression fit without using month 1 and month 2. The results remained statistically significant for improvement in the (1.4 % increase / month; [*p* = 0.0072] and 95 % confidence interval [0.67–2.19]).

## Discussion

The main finding in this study is that the combination of large numbers of observations with service-specific public feedback dramatically altered physician behavior, and that this was sustained for 2 years after the intervention. We found that the large magnitude of observations was convincing and compelling to physicians when discussing hand hygiene compliance by a given service. Measuring hand hygiene compliance at room entry and exit simplifies the World Health Organization’s 5 Moments for Hand Hygiene to just 2 moments: entering and exiting this patient’s zone. Therefore direct comparisons to compliance measured in the standard way taking into account all 5 moments cannot be done. The BUGG study published in 2013 in JAMA, wherein 20 US medical and SICUs participated in a cluster randomized trial of universal precautions for all patient contact, measured compliance on room entry and exit [15]. Hand hygiene rates in this study of more than 26,120 patients were ranged from as low as 50.2 % to a high of 78.3 % [15]. Another study evaluated a quality improvement method for increasing hand hygiene compliance at room entry or exit from baseline of 47.5 % at baseline to 81.0 % at study end [16]. Measuring hand hygiene in this manner was important because it was directly measuring adherence to our own hospital policy. Measuring hand hygiene as clinicians enter and exit a room is a practical application that preserves patient’s privacy, and has precedent in the literature.

We found that the comparative nature of reporting with report cards though not explicitly judgmental, triggered a competitive response with every service demonstrating improved hand hygiene compliance over the study period. Feedback with report cards stopped after the study period, yet to our surprise hand hygiene compliance was sustained at 94 %, and never once fell below 90 %, in the 24 months following the study. We did not try to correlate this with a decrease in infection since our SICU already had an extremely low incidence of MRSA infection or bacteremia due to horizontal infection control measures [17]. At this point, after more than 2 years since the intervention period was completed, it appears an enduring culture change has taken place with regard to physician behavior toward hand hygiene compliance.

Our findings demonstrated a greater improvement when compared with Measuring hand hygiene at room entry and exit, a European trial of active surveillance for antimicrobial-resistant bacteria reported in 2014 an improvement in mean hand hygiene compliance from 52–77 % [18]. It is clear that most hospitals worldwide continue to struggle with attaining and sustaining very high rates of hand hygiene compliance at room entry and exit.

There are several explanations as to why hand hygiene compliance of physicians improved over the study period. Wide internal distribution of the study results allowed passive public humiliation for underperforming services. Every month the report card resembling Fig. 2 was disseminated by email to all providers. It was clearly evident where each group of providers ranked both in relation to the 90 % compliance goal and in comparison to other services or specialties. Without naming specific individuals, but by delineating specific services, an element of public shame was an important and compelling ingredient in this program’s success. The knowledge that continual observations were being performed in the unit likely contributed to improvement with a powerful Hawthorne Effect, which has been shown specifically to increase hand hygiene compliance [19]. In this case, the Hawthorne Effect should not be seen as a confounder of the hand hygiene compliance but rather as a key and sustainable contributor to performance improvement.

The extremely large quantity of observations was another convincing ingredient; physicians are data-driven and because of the large sampling had confidence in the accuracy of the compliance measurements. The identification of individual services translated into undeniable accountability. Chiefs of service could request compliance rates broken down by subgroups (medical student, intern, resident, fellow, physician assistant and attending physician) within their specialty at any time. A representative but de-identified breakdown is shown in Table 1. This allowed some services to focus interventions on the least compliant members of their team.

**Table 1:** De-identified compliance of different members of sample specialty. These were available upon request to chiefs of service

Team member	Compliant encounters	Noncompliant encounters	Percent compliance
Attending physician	3	4	43 %
House officer	51	37	58 %
Physician assistant	59	26	69 %
Medical student	19	10	66 %
Totals	132	77	63 %

Some authors have recommended motivation of appropriate hand hygiene practices through role modeling and peer pressure from senior medical, nursing and administrative staff [20]. It is likely that pressure from chiefs of service as well as peers, both of whom viewed the hand hygiene data, contributed to gains in compliance. More research is needed to elucidate the importance of social influence as a determinant of behavior change [21, 22].

There were unintended benefits to charging the unit coordinator with collection of hand hygiene data. During the transition to electronic medical records, there was concern from the unit coordinators about their job security, as their primary duties were to maintain the paper medical record. Our unit coordinator embraced this new responsibility, appreciated the opportunity to contribute in new ways, and reported higher job satisfaction knowing that her work was having a positive clinical effect. Unexpectedly, we observed a culture change in the SICU, a generalized empowerment of providers at all levels to be involved in the promotion of hand hygiene compliance; even medical students were observed to stop and remind attending physicians to “pump in and pump out”, a practice that might previously have been considered taboo in the hierarchical medical structure. Due to the meaningful use incentive program funded by the American Recovery and Reinvestment Act of 2009, hospitals across the country are racing to implement electronic health records [23]. These changes allow for opportunities in the shifting duties of unit coordinators who were previously needed for the maintenance of the paper medical record.

### Limitations

This study took place in a relatively short period of time in a single ICU with no control group. The entire sampling method is based on the reliability of one observer who was trained to make these observations according to written guidelines. There was no specific reliability testing or validation of the unit coordinator’s observations. The results of this study are subject to measurement bias. Feasibility of implementing a program of high-intensity observations may limit the external validity of this method, as some hospitals may lack the physical layout conducive to observations from the unit coordinator position. Unit coordinators in settings without electronic medical records may already have too many job responsibilities to allow time to gather such a large number of measurements. It could be argued that this is not a low-cost intervention because the unit coordinator could have been assigned other tasks vital to the functioning of that unit rather than data collection, or significant cost savings could have been obtained by reducing or eliminating the position. Alternatively, one should consider the annual cost savings of $39,500 from decreases in MRSA infection attributed to a 1 % increase in hand hygiene compliance for a 200-bed hospital [24]. We experienced no increase in cost at Tufts because our unit coordinator is a fixed rather than variable cost.

Because individuals were not named in the data collection, we cannot know whether some individuals were observed more than others, but assume this to be the case. Therefore, clustering, an event that occurs when some individuals have a disproportionate impact on results could have had an effect on our results. The very large numbers of observations should minimize this effect. Providers that washed their hands inside the private rooms with soap and water may have been falsely scored as noncompliant because the sinks may have been out of line of site of unit coordinator.

Due to the short nature of the study, we were unable to correlate changes in ICU-acquired infections in response to changes in hand hygiene compliance. Our methodology of a multiple intervention strategy does not allow for analysis of the effect of each individual component: known observer, the large number of observations, and monthly feedback. Instead, our method demonstrates that the paired intervention was highly effective at improving physician hand hygiene compliance.

## Conclusions

Physician hand hygiene compliance increased due to a paired strategy of measuring large numbers of hand hygiene observations by a known observer and publically providing a physician report card to service chiefs. Utilizing unit coordinators in this novel way can be part of an effective means to dramatically increase numbers of observations and increase hand hygiene compliance in a resource-limited environment. This strategy effectively changed the culture resulting in an unexpectedly sustained hand hygiene compliance rate long after monthly feedback was stopped.

## Key messages

- Physician hand hygiene compliance is frustratingly difficult to improve and maintain.
- Using the unit secretary to continually record hand hygiene observations can lead to large amounts of observations.
- Physicians responded with behavior change when confronted with specialty specific hand hygiene data, which was sustained long after intervention.

## Abbreviations

ABHR: alcohol-based hand rub
CPOE: computerized physician order entry
HCAI: healthcare-associated infections
HCWs: healthcare workers
MRSA: methicillin-resistant *Staphylococcus aureus*
SICU: surgical intensive care unit

## References

[1] Vincent JL, Rello J, Marshall J, Silva E, Anzueto A, Martin CD (2009). International study of the prevalence and outcomes of infection in intensive care units. JAMA.

[2] Ling ML, How KB (2012). Impact of a hospital-wide hand hygiene promotion strategy on healthcare-associated infections. Antimicrob Resist Infect Control.

[3] Kirkland KB, Homa KA, Lasky RA, Ptak JA, Taylor EA, Splaine ME (2012). Impact of a hospital-wide hand hygiene initiative on healthcare-associated infections: results of an interrupted time series. BMJ Qual Saf.

[4] Rosenthal VD, Guzman S, Safdar N (2005). Reduction in nosocomial infection with improved hand hygiene in intensive care units of a tertiary care hospital in Argentina. Am J Infect Control.

[5] Conly JM, Hill S, Ross J, Lertzman J, Louie TJ (1989). Handwashing practices in an intensive care unit: the effects of an educational program and its relationship to infection rates. Am J Infect Control.

[6] Boyce JM, Pittet D (2002). Guideline for hand hygiene in health-care settings. Am J Infect Control.

[7] WHO Guidelines on Hand Hygiene in Health Care: First Global Patient Safety Challenge Clean Care Is Safer Care. Geneva: World Health Organization; 2009. 23805438

[8] The Joint Commission: Center for Transforming Healthcare Content: Hand Hygiene Project. 2012. www.centerfortransforminghealthcare.org/projects/detail.aspx?Project=3 (last accessed November 15, 2012).

[9] Erasmus V, Daha TJ, Brug H, Richardus JH, Behrendt MD, Vos MC (2010). Systematic review of studies on compliance with hand hygiene guidelines in hospital care. Infect Control Hosp Epidemiol.

[10] Pittet D, Simon A, Hugonnet S, Pessoa-Silva CL, Sauvan V, Perneger TV (2004). Hand hygiene among physicians: performance, beliefs, and perceptions. Ann Intern Med.

[11] Biddle C, Shah J (2012). Quantification of anesthesia providers’ hand hygiene in a busy metropolitan operating room: what would Semmelweis think?. Am J Infect Control.

[12] Doron SI, Kifuji K, Hynes BT, Dunlop D, Lemon T, Hansjosten K (2011). A multifaceted approach to education, observation, and feedback in a successful hand hygiene campaign. Jt Comm J Qual Patient Saf.

[13] Reisinger HS, Yin J, Radonovich L, Knighton VT, Martinello RA, Hodgson MJ (2013). Comprehensive survey of hand hygiene measurement and improvement practices in the Veterans Health Administration. Am J Infect Control.

[14] Haas JP, Larson EL (2007). Measurement of compliance with hand hygiene. J Hosp Infect.

[15] Harris AD, Pineles L, Belton B, Johnson JK, Shardell M, Loeb M (2013). Universal glove and gown use and acquisition of antibiotic-resistant bacteria in the ICU: a randomized trial. JAMA.

[16] Chassin MR, Mayer C, Nether K (2015). Improving hand hygiene at eight hospitals in the United States by targeting specific causes of noncompliance. Jt Comm J Qual Patient Saf.

[17] Traa MXBL, Doron S, Syndman D, Noubary F, Nasraway SA (2014). Horizontal infection control strategy decreases methicillin-resistant Staphylococcus aureus infection and eliminates bacteremia in a surgical ICU without active surveillance. Crit Care Med.

[18] Derde LP, Cooper BS, Goossens H, Malhotra-Kumar S, Willems RJ, Gniadkowski M (2014). Interventions to reduce colonisation and transmission of antimicrobial-resistant bacteria in intensive care units: an interrupted time series study and cluster randomised trial. Lancet Infect Dis.

[19] Eckmanns T, Bessert J, Behnke M, Gastmeier P, Ruden H (2006). Compliance with antiseptic hand rub use in intensive care units: the Hawthorne effect. Infect Control Hosp Epidemiol.

[20] Whitby M, Pessoa-Silva CL, McLaws ML, Allegranzi B, Sax H, Larson E (2007). Behavioural considerations for hand hygiene practices: the basic building blocks. J Hosp Infect.

[21] Huis A, van Achterberg T, de Bruin M, Grol R, Schoonhoven L, Hulscher M (2012). A systematic review of hand hygiene improvement strategies: a behavioural approach. Implement Sci.

[22] Huis A, Schoonhoven L, Grol R, Donders R, Hulscher M, van Achterberg T (2013). Impact of a team and leaders-directed strategy to improve nurses’ adherence to hand hygiene guidelines: A cluster randomised trial. Int J Nurs Stud.

[23] Blumenthal D (2009). Stimulating the adoption of health information technology. N Engl J Med.

[24] Cummings K, Anderson D, Kaye K (2010). Hand hygiene noncompliance and the cost of hospital-acquired methicin-resistant Staphylococcus aureus infection. Infect Control Hosp Epidemiol.

